# Antibacterial activity of six medicinal Cameroonian plants against Gram-positive and Gram-negative multidrug resistant phenotypes

**DOI:** 10.1186/s12906-016-1371-y

**Published:** 2016-10-10

**Authors:** Igor K. Voukeng, Veronique P. Beng, Victor Kuete

**Affiliations:** 1 Department of Biochemistry, Faculty of Science, University of Dschang, P.O. Box 67, Dschang, Cameroon; 2 Department of Biochemistry, Faculty of Science, University of Yaounde I, Yaounde, Cameroon

**Keywords:** Cameroon, Gram-negative bacteria, Gram-positive bacteria, Medicinal plant, Multi-drug resistance, Antibacterial activity

## Abstract

**Background:**

Infectious diseases due to multidrug-resistant bacteria are one of the causes of treatment failures contributing to an increase in mortality and/or morbidity. In this study, we evaluated the antibacterial potential of different parts of six medicinal plants namely *Alstonia boonei, Ageratum conyzoides, Croton macrostachys, Cassia obtusifolia, Catharanthus roseus* and *Paullinia pinnata* against a panel of 36 multi-drug resistant (MDR) Gram-negative and Gram-positive bacteria.

**Methods:**

Minimum Inhibitory Concentration (MIC) and Minimal Bactericidal Concentration (MBC) of the methanol extracts from different parts of the plants were determined using broth microdilution method; standard phytochemical methods were used for phytochemical screening.

**Results:**

Several phytochemical classes such as polyphenols, sterols, triterpenes, alkaloids, flavonoids and saponins were identified in the plant extracts. MIC values obtained ranged from 64 to 1024 μg/mL. Leaves extract of *Catharanthus roseus* (86.11 %), *Croton macrostachys* (83.33 %) and *Paullinia pinnata* (80.55 %) displayed the best antibacterial spectra. The lowest MIC value of 64 μg/mL was obtained with the *Paullinia pinnata* stems extract and *Cassia obtusifolia* extract against the strain of *Staphylococcus aureus* MRSA8. Results also showed that the tested samples generally displayed bacteriostatic effects with MBC values obtained in only 3.35 % of the cases where plant extracts were active.

**Conclusion:**

The results obtained at the end of this study demonstrate for the first time the antibacterial activity of the studied medicinal plants against MDR bacteria. The tested plants could be a reservoir of molecules to fight against MDR bacterial infections.

## Background

Infectious diseases caused by multidrug-resistant bacteria are growing steadily and are associated with a significant attributable mortality [1, 2]. The emergence of multi-drug resistant (MDR) phenotypes was first linked to nosocomial infections; but nowadays they are increasingly responsible for community infections and all pathogenic microorganisms are concerned. In Gram-negative bacteria, one of the mechanisms of resistance is the lowering of intracellular amount of antibacterial substances due to the presence of the resistance nodulation cell division (RND)-type efflux pumps. This phenomenon gives possibility to bacteria developing resistance to a wide range of antibiotics, as well as several biocides [3, 4]. Gram-positive bacteria are also a major cause of hospitalization; infections due to *Staphylococcus aureus* resistant to methicillin (MRSA) are a major health problem both in hospitals and community environments [5]. MRSA is responsible for 80461 severe infections and causing the death of 11,285 patients annually in the United States [6]. One of the possible ways to overcome this phenomenon of multi-resistance is the continual search for new antibacterial molecules active vis-à-vis of MDR bacteria. With regard to the broad diversity of their secondary metabolites, medicinal plants represent undeniable sources of antibacterial agents. According to WHO [7], 80 % of people in Africa have used medicinal plants for their health care; it is also estimated that among medicines sold worldwide, 30 % contain compounds derived from medicinal plants [8]. Several African medicinal plants previously investigated for biological potential showed good antibacterial activities. Some of them include *Treculia obovoidea* [9], Albi*zia adianthifolia Laportea ovalifolia* [10], *Alchornea cordifolia*, *Pennisetum purpureum* [11]. In our continuous search of phytochemicals to combat MDR bacterial infections, we designed the present study to evaluate the antimicrobial potential of six Cameroonian medicinal plants namely *Alstonia boonei, Catharanthus roseus, Ageratum conyzoides, Croton macrostachys, Cassia obtusifolia,* and *Paullinia pinnata* vis-à-vis MDR Gram-negative and Gram-positive phenotypes.

## Methods

### Plant materials and extraction

Various parts of plant (Table 1) were collected from different regions in Cameroon during the month of February 2014. These include *Alstonia boonei* (leaves and bark)*, Catharanthus roseus* (leaves and stem)*, Ageratum conyzoides* (whole plant)*, Croton macrostachys* (leaves)*, Cassia obtusifolia* (whole plant)*,* and *Paullinia pinnata* (leaves and stem). After drying, each part was powdered and soaked in methanol for 48 h at room temperature, and then filtered using Whatman filter paper N°1. The filtrate obtain were concentrated at 50 °C under reduce pressure in a vacuum to obtain each plant extract.

**Table 1:** Information on plant used in the present study

Plant family/Plant sample - Herbarium voucher number	Traditional use	Part used in this study	Potential active compounds characterized	Previously screened activity
APOCYNACEAE*/Alstonia boonei* De Wild – 43368/HNC	Fever, painful micturition, insomnia, chronic diarrhea, rheumatic pains, anti-venom (snake bites), malaria, diabetes, helminths, arthritis [28, 29].	Leaves, bark	Echitamine, echitamidine, Voacangine, akuammidine, N- *α*-formylechitamidine, N*- α*-formyl-12-methoxyechitamidine [29].	Antimalarial, antioxidant, analgesic, anti-inflammatory, antipyretic [30–32].
*APOCYNACEAE/Catharanthus roseus* L. – 5689/HNC.	Bleeding arresting, diabetes, fever, rheumatism, cancer [20, 33].	Leaves, stem	Vincristine, vinblastine, benzoic acid, p-hydroxybenzoic acid, salicylic acid, 2,3-dihydroxybenzoic acid, 2,5-dihydroxybenzoic acid, 3,4-dihydroxybenzoic acid, 3,5-dihydroxybenzoic acid, gallic acid, vanillic acid, chlorogenic acid, kaemferol trisaccharides, Quercetin trisaccharides, Syringetin glycosides [20, 34].	Wound-healing, antimicrobial, hypoglycemic, antioxidant [18, 20, 33].
ASTERACEAE*/Ageratum conyzoïdes* Linn. – 19050/SFR-Cam	Purgative, fever, ulcers, wound, mental, infectious diseases, headaches, anti-inflammatory, diarrhea [35, 36].	Whole plant	*β*-caryophyllene, precocene I, friedelin, Lycopsamine, echinatine,*β*-sitosterol, stigmasterol, 5-methoxynobiletin, linderoflavone B, eupalestin, sabinene, *α* and *β* pinene, *β*-phellandrene, 1,8-cineole and limonene, ocimene, eugenol [35].	Antimicrobial, anticonvulsant, analgesic, anti-inflammatory, antipyretic, insecticidal, antioxidant, antiplasmodial, cytotoxic [35, 37, 38].
EUPHORBIACEAE*/Croton macrostachys* Hochst. – 40501/HNC	Malaria, antidiabetic, purgative mastitis, wounds, gastrointestinal Complications [39–41].	Leaves	Neoclerodan-5,10-en-19,6*β*;20,12-diolide; 3*α*,19-Dihydroxytrachylobane; 3*α*,18,19-Trihydroxytrachylobane, lupeol, lupenone, betulinic acid, 28-*O*-acetylbetulin, betulin, lupeol acetate, zeorin, benzoic acid, methyl gallate, methyl 2,4-dihydroxy-3,6-dimethylbenzoate, lichexanthone, *β*-sitosterol, and *β*-sitosterol palmitate, stigmasterol, botulin, crotepoxide [42, 43].	Antimicrobial, antimalarial, cytotoxic [38, 39, 41].
FABACEAE*/Cassia obtusifolia* L. – 39847/HNC	Laxative, eye infections, diarrhea, urinary tract infections, gingivitis, fever, cough [25].	Whole plant	aloe-emodin, 1-methylaurantio-obtusin-2-*O-β*-_D_-glucopyranoside, emodin, 1,2- dihydroxyanthraquinone, obtusin, chrysoobtusin, aurantioobtusin, gluco-obtusifolin, glucoaurantioobtusin, gluco-chryso-obtusin, 1-desmethylaurantio-obtusin, 1-desmethylaurantio-obtusin-2-*O-β*- _D_-glucopyranoside, 1-desmethylchryso-obtusin, 1-desmethyl-obtusin, aurantio-obtusin-6-*O-β*-_D_-glucopyranoside, alaternin-1-*O-β*-_D_-glucopyranoside, chrysoobtusin-2-*O-β*-_D_-glucopyranoside physicon-8-*O-β*-_D_-glucoside, obtusifolin, *O*-methyl-chrysophanol, emodin-1-*O-β*-gentio-bioside, chrysophanol-1-*O-β*-gentiobioside, physcion-8-O-β-gentiobioside, physcion-8-*O-β*-glucoside, chrysophanol-1-*O-β*-_D_-glucopyranosyl-(13)-*β*-_D_-glucopyranosyl-(1 → 6)-*β*-_D_-glucopyranoside, chrysophanic acid, physcion, questin, 1,3-dihydroxy-8-methylanthraquinone, chrysophanol- 10,10′-bianthrone, torosachrysone [44].	Antibacterial, antifungal, mosquito larvicidal activity, platelet antiaggregatory, neuroprotective [25, 45–47].
SAPINDACEAE*/Paullinia pinnata* L. – 10702/SRF-Cam	Malaria, erectile dysfunction [24].	Leaves, stem	Paullinoside A, paullinomides A and B, *β*-amyrin, 13β,17*β*-dihydroxy-28-norolean-12-ene, *β*-sitosterol glucopyranoside, 2-*O*-methyl-L-chiro-inositol, L-quebrachitol, *β*-sitosterol, friedelin, daucosterol, aridanin, lotoidoside [24, 48].	Antiparasitic, antimicrobial, cytotoxic [24, 38, 49].

*HNC* Cameroon National Herbarium, *SRF-Cam* Société’ des Réserves Forestières du Cameroun

### Preliminary phytochemical screenings

The presence of alkaloids, triterpenes, sterols, flavonoids, polyphenols and saponins were screened according to the common phytochemical methods described by Harborne [12].

### Chemicals

Chloramphenicol and ciprofloxacin (Sigma–Aldrich, St. Quentin Fallavier, France) were used as reference antibiotics meanwhile *p*-Iodonitrotetrazolium chloride (INT) was used as microbial growth indicator.

### Bacterial strains and culture media

The studied microorganisms included ATCC (American Type Culture Collection) and MDR clinical strains of Gram-negative bacteria (*Escherichia coli, Pseudomonas aeruginosa, Enterobacter aerogenes, Providencia stuartii, Klebsiella pneumoniae* and *Enterobacter cloacae*) and Gram-positive bacteria (*Staphyloccocus aureus*). Their bacterial features are summarized in Table 2; they were maintained at 4 °C on McConkey agar and Mannitol Salt Agar (MSA) for Gram negative and Gram positive bacteria respectively, and sub-cultured on Mueller Hinton Agar (MHA) for 24 h before any test. Mueller Hinton Broth (MHB) was used for MIC and MBC determinations.

**Table 2:** Bacterial strains used in this study and their features

Strains	Characteristics	References
*Escherichia coli*		
ATCC10536	Reference strain	
AG100	Wild-type *E. coli* K-12	[50]
AG100A	AG100 *ΔacrAB*::KAN^R^	
AG100A_TET_	Δ*acrAB* mutant AG100, with over-expressing *acrF* gene ; TET^R^	[50–52]
AG102	Δ*acrAB* mutant AG100, owing *acrF* gene markedly over-expressed; TET^R^	[53, 54]
MC4100	Wild type *E. coli*	[55]
W3110	Wild type *E. coli*	[55, 56]
*Enterobacter aerogenes*		
ATCC13048	Reference strain	
CM64	CHL^R^ resistant variant obtained from ATCC13048 over-expressing the AcrAB pump	[57]
EA3	Clinical MDR isolate; CHL^R^, NOR^R^, OFX^R^, SPX^R^, MOX^R^, CFT^R^, ATM^R^, FEP^R^	[58, 59]
EA27	Clinical MDR isolate exhibiting energy-dependent norfloxacin and chloramphenicol efflux with KAN^R^ AMP^R^ NAL^R^ STR^R^ TET^R^	[58, 59]
EA289	KAN sensitive derivative of EA27	[60]
EA294	EA289 a*crA::*KAN^R^	[60]
EA298	EA 289 *tolC::*KAN^R^	[60]
*Enterobacter cloacae*		
ECCI69	Clinical MDR isolates, CHL^R^	[61]
BM67	Clinical MDR isolates, CHL^R^	[61]
BM47	Clinical MDR isolates, CHL^R^	[61]
*Klebsiella pneumoniae*		
ATCC12296	Reference strain	
KP55	Clinical MDR isolate, TET^R^, AMP^R^, ATM^R^, CEF^R^	[62]
KP63	Clinical MDR isolate, TET^R,^ CHL^R^, AMP^R,^ ATM^R^	[62]
K24	AcrAB-TolC, Laboratory collection of UNR-MD1, University of Marseille, France	[61]
K2	AcrAB-TolC, Laboratory collection of UNR-MD1, University of Marseille, France	[61]
*Providencia stuartii*		
NEA16	Clinical MDR isolate, AcrAB-TolC	[63]
ATCC29916	Clinical MDR isolate, AcrAB-TolC	
PS2636	Clinical MDR isolate, AcrAB-TolC	
PS299645	Clinical MDR isolate, AcrAB-TolC	
*Pseudemonas aeruginosa*		
PA 01	Reference strain	
PA 124	MDR clinical isolate	[64]
S. aureus		
ATCC 25923	Reference strain	
MRSA 3	Clinical MDR isolate OFX^R^, KAN^R^, TET^R^, ERM^R^	[65]
MRSA 4	Clinical MDR isolate OFX^R^, KAN^R^, CHL^R^, CIP^R^	
MRSA 6	Clinical MDR isolate OFX^R^, FLX^R^, KAN^R^, TET^R^, CIP^R^, IM/CS^R^	
MRSA 8	Clinical MDR isolate OFX^R^, FLX^R^, KAN^R^, ERM^R^, CIP^R^, IM/CS^R^	
MRSA 11	Clinical MDR isolate OFX^R^, KAN^R^, ERM^R^, CIP^R^, IM/CS^R^	
MRSA 12	Clinical MDR isolate OFX^R^, FLX^R^, KAN^R^, ERM^R^, IM/CS^R^	

AMP^R^, ATM^R^, CEF^R^, CFT^R^, CHL^R^, CIP^R^, ERM^R^, FEP^R^, FLX^R^, IM/CS^R^, KAN^R^, MOX^R^, OFX^R^, STR^R^, TET^R^, Resistance to ampicillin, aztreonam, cephalothin, cefadroxil, chloramphenicol, Ciprofloxacin, Erythromycin, cefepime, Flomoxef, Imipenem/Cilastatin sodium, kanamycin, moxalactam, streptomycin, and tetracycline; *MDR* multidrug resistant

### INT colorimetric assay for MIC and MBC determinations

Minimal inhibitory concentrations (MIC) of different plant extracts were determined using broth microdilution method described by Kuete et al. [13] with some modifications [9]. Briefly, plant extracts, chloramphenicol and ciprofloxacin were dissolved in dimethylsufoxide (DMSO)-MHB (10:90) and 100 μL each solution was added to a 96 wells microplate containing MHB, then serially diluted two-fold, followed by adding of 100 μL of inoculum prepared in MHB. The microplate was sealed and incubated for 18 h at 37 °C. The final concentration of inoculum was 1.5 ×10^6^ CFU/mL and less than 2.5 % for DMSO in each well; Wells containing DMSO 2.5 % and inoculums were used as negative control whereas chloramphenicol and ciprofloxacin consist of positive control. After 18 h incubation, 40 μL of INT (0.2 mg/mL) was added to each well and re-incubated for 30 min. MIC was defined as the lowest concentration of plant extract that inhibited bacterial growth.

The determination of MBC was made by introducing 150 μL of MHB in each well of 96 well plate. Then 50 μL of the well contents which did not show any growth after incubation during MIC assays was introduced in the aforesaid plate accordingly, and incubated at 37 °C for 48 h. The MBC was defined as the lowest concentration of plant extract, which did not produce a color change after addition of INT as described previously.

## Results

### Phytochemical composition

The results of qualitative analysis (Table 3) showed that plant extracts contain various phytochemical classes of secondary metabolites. Polyphenols, triterpenes and saponins were present in all plant extracts except those from *Cassia obtusifolia, Catharanthus roseus* leaves and stem respectively.

**Table 3:** Extraction yields and phytochemical composition of the plant extracts

Plant extract (used part)	Extraction yield (%)	Phytochemicals groups					
		Alkaloids	Triterpenes	Sterols	Flavonoids	Polyphenols	Saponins
*A. boonei* (leaves)	15.8 %	-	-	+	-	+	+
*A. boonei* (bark)	9.65 %	+	+	+	+	+	+
*A. conyzoïdes* (whole plant)	8.52 %	-	+	+	-	-	+
*C. macrostachys* (Leaves)	12.72 %	-	+	+	-	+	+
*C. obtusifolia* (whole plant)	7.11 %	+	+	+	+	+	+
*C. roseus* (leaves)	6.89 %	+	-	+	+	+	+
*C. roseus* (stem)	4.23 %	+	+	+	+	+	-
*P. pinnata* (leaves)	10.84 %	-	+	+	-	+	+
*P. pinnata* (stem)	5.47 %	-	+	+	-	+	+

+: presence; −: absence

### In vitro antibacterial effect of plant extract

The methanol extracts from different parts of plants were tested on 36 bacterial strains including 7 Gram-positive and 29 Gram-negative bacterial strains. As shown in Table 4, extracts from leaves of *Alstonia bonnei, Paullinia pinnata* and *Catharanthus roseus* displayed wide spectra of activity in comparison to those from bark and stems of the same plants. The various plant extracts (when they were active) had MIC between 64 and 1024 μg/mL. Leaves of *Catharanthus roseus* showed the best spectrum of activity, inhibiting the growth of 86.11 % (31/36) of the bacteria (24/29 Gram-negative bacteria and 7/7 Gram-positive bacteria). The leaves extract of *Croton macrostachys* also had an interesting activity (30/36; 83.33 %), followed by extract of the leaves of *P. pinnata* (29/36; 80.55 %) and the whole plant extract of *A. conyzoides* (25/36; 69.44 %). The lowest MIC value of 64 μg/mL was obtained with the *Paullinia pinnata* stems extract and *Cassia obtusifolia* extract against the strain of *Staphylococcus aureus* MRSA8. In general, analysis of results shows that MBCs were obtained in 3.35 % (7/209) of cases where plant extracts were active.

**Table 4:** MIC and MBC (in bracket) of plant extracts and reference drugs

	*A. conyzoïdes* (whole plant)	*A. boonei*		*C. obtusifolia* (whole plant)	*C. roseus*		*C. macrostachys* (leaves)	*P. pinnata*		Reference drugs
		Leaves	Bark		Leaves	Stem		Leaves	(Stem)	Chloramphenicol
*Escherichia coli*										
ATCC8739	-	512 (−)	-	-	512 (−)	-	512 (−)	1024 (−)	-	2 (128)
ATCC10536	256 (−)	-	-	-	512 (−)	1024 (−)	256 (−)	128 (−)	-	<2 (64)
AG100	1024 (−)	-	-	256 (1024)	1024 (−)	-	1024 (−)	256 (−)	128 (−)	8 (128)
AG100A	1024 (−)	-	-	512 (−)	128 (−)	256 (−)	-	256 (−)	256 (−)	<2 (128)
AG100A_TET_	1024 (−)	512 (−)	1024 (−)	-	1024 (−)	-	-	-	512 (−)	32 (−)
AG102	512 (−)	512 (−)	1024 (−)	-	-	-	1024 (−)	-	256 (−)	64 (−)
MC4100	-	-	-	-	512 (−)	512 (−)	256 (−)	256 (−)	1024 (−)	16 (−)
W311O	1024 (−)	-	128 (−)	-	512 (−)	-	256 (−)	1024 (−)	-	2 (−)
*Pseudomonas aeruginosa*										
PA 01	-	-	-	256 (−)	512 (−)	256 (−)	256 (−)	256 (−)	1024 (−)	32 (−)
PA 124	-	-	-	-	-	-	-	-	-	128 (−)
*Enterobacter aerogenes*										
ATCC13048	1024 (−)	512 (−)	-	512 (−)	-	-	128 (−)	-	-	4 (32)
EA-CM64	256 (−)	-	-	512 (−)	1024 (−)	-	256 (−)	512 (−)	1024 (−)	256 (−)
EA3	-	-	-	-	256 (−)	1024 (−)	128 (−)	-	-	256 (−)
EA27	256 (−)	512 (−)	512 (−)	512 (1024)	512 (−)	1024 (−)	512 (−)	512 (−)	-	32 (−)
EA289	512 (−)	1024 (−)	1024 (−)	256 (−)	512 (−)	1024 (−)	512 (−)	512 (−)	512 (−)	64 (−)
EA298	1024 (−)	-	1024 (−)	512 (−)	1024 (−)	1024 (−)	128 (−)	512 (−)	512 (−)	128
*Providencia stuartii*										
NEA16	1024 (−)	512 (−)	1024 (−)	1024	1024 (−)	-	1024 (−)	1024 (−)	1024 (−)	32 (256)
ATCC29916	512 (−)	512 (−)	-	-	-	-	256 (−)	1024 (−)	-	16 (256)
PS2636	256 (−)	-	-	-	-	256 (−)	256 (−)	256 (−)	-	16 (256)
PS299645	1024 (−)	512 (−)	-	-	256 (−)	512 (−)	512 (−)	512 (−)	-	64 (−)
*Klebsiella pneumoniae*										
ATCC11296	512 (−)	512 (−)	1024 (−)	1024 (−)	1024 (−)	1024 (−)	512 (−)	1024 (−)	-	8 (256)
KP55	512 (−)	512 (−)	-	256 (−)	512 (−)	-	256 (−)	1024 (−)	256 (−)	32 (256)
KP63	1024 (−)	1024 (−)	-	1024 (−)	512 (−)	-	-	1024 (−)	1024 (−)	32 (−)
K24	1024 (−)	512 (−)	1024 (−)	1024 (−)	1024 (−)	-	512 (−)	512 (−)	-	64 (256)
K2	1024 (−)	256 (−)	-	1024 (−)	512 (−)	512 (−)	-	-	-	8 (256)
*Enterobacter cloacae*										
ECCI69	-	1024 (−)	1024 (−)	1024 (−)	1024 (−)	-	1024 (−)	512 (−)	1024 (−)	-
BM47	-	-	-	-	1024 (−)	-	512 (−)	1024 (−)	1024 (−)	256 (−)
BM67	512 (−)	512 (−)	1024 (−)	512 (−)	256 (−)	-	256 (−)	1024 (−)	-	-
BM94	1024 (−)	512 (−)	1024 (−)	512 (−)	512 (−)	-	512 (−)	1024 (−)	-	128 (−)
*Staphyloccocus aureus*										Ciprofloxacin
ATCC25923	512 (−)	256 (−)	-	256 (1024)	512 (−)	1024 (−)	256 (−)	256 (−)	128 (1024)	2 (8)
MRSA 3	-	-	-	-	1024 (−)	-	-	-	-	32 (128)
MRSA 4	256 (−)	256 (−)	-	128 (1024)	512 (−)	-	256 (−)	256 (−)	128 (512)	64 (128)
MRSA 6	-	128 (−)	-	256 (−)	1024 (−)	512 (−)	512 (−)	256 (−)	256 (−)	64 (128)
MRSA 8	-	128 (−)	-	64 (512)	128 (−)	1024 (−)	512 (−)	256 (−)	64 (512)	16 (64)
MRSA 11	1024 (−)	-	-	512 (−)	1024 (−)	1024 (−)	1024 (−)	512 (−)	512 (−)	128 (256)
MRSA 12	-	128 (−)	-	256 (−)	1024 (−)	1024 (−)	512 (−)	256 (−)	256 (−)	32 (32)

(−): MIC or MBC not detected up to 1024 μg/mL for plant extracts and 256 μg/mL for reference drugs

## Discussion

Several classes of secondary metabolites such as alkaloids, triterpenes, sterols, flavonoids, polyphenols and saponins have been reported to have antibacterial properties [13–15]. Their presence in the studied plant extracts could explain the antibacterial effects of the tested samples. The need to find new molecules from medicinal plants with effective mechanisms of action against the multidrug-resistant phenotype is a necessity nowadays. All plants used in traditional medicine which have MIC values less than 8 mg/mL are considered active [16]. A plant extract has significant antibacterial activity if MIC is ˂100 μg/mL, moderate if its MIC is between 100 and 625 μg/mL and low when MIC is above 625 μg/mL [17]. Based on the above criteria, it can be deduced that all tested plants had antibacterial activity as MIC values below 8 mg/mL were obtained with each extract on at least one bacterial strain. MIC values above 625 μg/mL were obtained with extract from *A. boonei* bark against 2/36 (5.5 %) tested bacteria as well as with *C. roseus* stem extract against 6/36 (16.7 %) microorganisms tested, indicating that they rather displayed low antibacterial effects. Nonetheless, the activity obtained with the *Paullinia pinnata* stems extract and *Cassia obtusifolia* extract against the strain of *Staphylococcus aureus* MRSA8 (MIC value of 64 μg/mL) could be considered important. Moderate activity was obtained in many cases. In fact, MIC values ranged from 128 to 512 μg/mL were obtained with extract from *A. conyzoides* (whole plant) against 12/36 (33.3 %) tested bacteria*, A. boonei leaves* against 19/36 (52.8 %), *C. obtusifolia* (whole plant) against 17/36 (47.2 %), *C. roseus* leaves against 18/36 (50 %), *C. macrostachys* (leaves) against 25/36 (69.4 %), and *P. pinnata* stem and leaves against 13/36 (36.1 %) and 19/36 (52.8 %) respectively.

Though the antibacterial activities of some of the tested plants have already been reported, their effects against MDR phenotypes are being documented for the first time. The extract from the leaves of *C. roseus* had a broad antibacterial activity (31/36; 86.11 %); Nayak and Pereira [18] and Kamaraj et al. [19] reported the antibacterial activity of this plant extract on some sensitive bacteria. Several alkaloids were isolated from this plant [20, 21] and these compounds could also be responsible for the antibacterial activity of this plant [22]. MIC values obtained with extract of leaves of *C. macrostachys* are between 128 and 1024 μg/mL; Antibacterial compounds previously isolated from this plant include the triterpenoid, lupeol [23]. The extract of *P. pinnata* possessed a good activity (MIC of 64 μg/mL) against *S. aureus* MRSA8 while the extract from the leaves was active against 80.55 % (29/36) of the studied microorganisms. Lunga et al. [24] demonstrated the activity of this plant on strains of *Salmonella sp*. with a bacteriostatic effect, corroborating our findings. The extract of *C. obtusifolia* significantly inhibited the growth of *S. aureus* MRSA8 with MIC of 64 μg/mL, and was active on 22 of the 36 tested microorganisms. The activity obtained in this study is much better than that mentioned by Doughari et al. [25]. In fact, they obtained MIC of 2000 μg/mL and 1000 μg/mL on clinical isolate of *S. aureus* and *P. aeruginosa* respectively. This could be due to the difference of phytochemical composition as the environmental conditions influence the availability as well as the amounts of some secondary metabolites in the plant. One of the best suited secondary metabolite from this plant is emodin (anthraquinone) which possesses a good antibacterial activity against *S. aureus* [26]; this could explain the interesting activity observed vis-à-vis of MRSA in this study. The extract of *A. conyzoides* had a relatively low activity on all studied microorganisms. Nevertheless, MIC of 256 μg/mL vis-a-vis *E. aerogenes* EA-CM64 and EA27, *P. stuartii* PS2636, *S. aureus* MRSA 4 which are multi-drug resistant clinical strains were obtained; this could explain the use of this plant in traditional medicine. Leaves and bark extracts of *A. bonnei* had a moderate activity against Gram-negative bacteria whilst bark extract was not active against Gram-positive species; this is explained by the fact that some antimicrobial compounds have specific activity spectrum (narrow) and therefore will not be active on certain categories or certain species of microorganisms [27]. Though the overall activity of the tested plants can be considered moderate, the results of this study are interesting taking in account the fact that most of the tested bacterial strains were MDR phenotypes.

## Conclusion

The present study demonstrates that plants studied and mostly *C. macrostachys*, *C. roseus* and *P. pinnata* contain phytochemicals with valuable antibacterial activities vis-à-vis multi-drug resistant phenotypes. They could be used in the management of bacterial infections including MDR phenotypes.

## Abbreviations

*A. conyzoides*: *Ageratum conyzoides*
*Alstonia boonei*: *Alstonia boonei*
ATCC: American type culture collection
*C. macrostachys*: *Croton macrostachys*
*C. roseus*: *Catharanthus roseus*
*Cassia obtusifolia*: *Cassia obtusifolia*
CFU: Colony forming unit
DMSO: Dimethylsufoxide
*E. aerogenes*: *Enterobacter aerogenes*
*E. cloacae*: *Enterobacter cloacae*
*E. coli*: *Escherichia coli*
INT: *p*-Iodonitrotetrazolium chloride
*K. pneumoniae*: *Klebsiella pneumoniae*
MBC: Minimal bactericidal concentration
MDR: Multi-drug resistant
MHA: Mueller Hinton Agar
MHB: Mueller Hinton Broth
MIC: Minimum inhibitory concentration
MRSA: Methicillin resistant *Staphylococcus aureus*
MSA: Mannitol Salt Agar
*P. aeruginosa*: *Pseudomonas aeruginosa*
*P. pinnata*: *Paullinia pinnata*
*P. stuartii*: *Providencia stuartii*
RND: Resistance nodulation cell division
*S. aureus*: *Staphyloccocus aureus*
